# The association of preprocedural C-reactive protein/albumin ratio with in-stent restenosis in patients undergoing iliac artery stenting

**DOI:** 10.34172/jcvtr.2020.31

**Published:** 2020-08-16

**Authors:** Ali Nazmi Çalık, Duygu İnan, Mehmet Baran Karataş, Evliya Akdeniz, Duygu Genç, Yiğit Çanga, Tufan Çınar, Ayşe Emre

**Affiliations:** ^1^University of Health Sciences, Dr. Siyami Ersek Thoracic and Cardiovascular Surgery Training and Research Hospital, Istanbul, Turkey; ^2^University of Health Sciences, Sultan Abdülhamid Han Training and Research Hospital, Istanbul, Turkey

**Keywords:** C-Reactive Protein, Albumin, Ratio, In-stent Restenosis, Iliac Artery Disease

## Abstract

***Introduction:*** In-stent restenosis (ISR) still constitutes a major problem after percutaneous vascular interventions and the inflammation has a pivotal role in the pathogenesis of such event. The C-reactive protein/albumin ratio (CAR) is a newly identified inflammatory biomarker, and it may be used as an indicator to predict ISR in subjects with coronary artery stenting. In light of these data, our main objective was to investigate the relationship between the preprocedural CAR and ISR in patients undergoing successful iliac artery stent implantation.

***Methods:*** In total, 138 consecutive patients who had successful iliac artery stent implantation in a tertiary heart center between 2015 and 2018 were enrolled in the study. The study population was categorized into two groups; patients with ISR and those without ISR during follow-up. The CAR was determined by dividing CRP by serum albumin.

***Results:*** In the multivariable regression analysis; the CAR (HR: 2.66, 95% CI: 1.66-4.25, *P* < 0.01), stent length (HR: 1.01, 95% CI: 0.99-1.02, *P* = 0.04), and HbA1c levels (HR: 1.22, 95% CI: 0.99-1.51, *P* = 0.04) were independently related with ISR. A receiver operating curve analysis displayed that the CAR value of >0.29 predicted ISR with sensitivity of 97.5% and specificity of 88.8% (AUC 0.94, *P* < 0.01).

***Conclusion:*** Our findings provide evidence that the CAR may be an applicable inflammatory biomarker in predicting ISR in subjects undergoing iliac artery stenting for the treatment of peripheral artery disease (PAD). Also, the stent length and poor glycemic control were found to be associated with ISR.

## Introduction

Peripheral artery disease (PAD) usually results from the atherosclerotic narrowing and thrombotic occlusion of the lower extremity arteries.^1^ Atherosclerotic involvement of infrarenal aorta and iliac arteries, which eventually causes aortoiliac occlusive disease (AIOD), constitutes approximately one-third of all PAD patients. Over the last few decades, endovascular techniques for AIOD have increasingly improved, resulting in a significant shift from open surgical procedures toward less-invasive percutaneous interventions, including balloon angioplasty and stenting.^2^ However, despite significant advances in stent technology, in-stent restenosis (ISR) remains a potential problem, particularly during the long-term follow-up period.

C-reactive protein (CRP) is an established systemic inflammatory biomarker, and it is a positive acute-phase reactant. By contrast, serum albumin is a negative phase reactant, which tends to decrease in response to the inflammatory response. It is well known that inflammation plays a pivotal role in the pathogenesis of ISR.^3^ Previous studies have demonstrated that both serum CRP and albumin levels are important inflammatory parameters for ISR in patients with coronary artery stenting.^4-6^ However, recent research has revealed that the combination of these parameters into a single index has yielded a better reflection of the inflammatory status and ISR development compared to serum CRP or albumin alone among these patients.^7^ Within this scope, the CRP/albumin ratio (CAR) can be suggested as a more sensitive marker of the inflammatory process and progression of the disease. On the other hand, in the current literature, there is a shortage of data with respect to the relation of CAR with ISR in patients undergoing iliac artery stenting. Therefore, our main aim was to investigate the relationship between the preprocedural CAR and ISR development in subjects with aortoiliac disease undergoing a stenting. To our knowledge, this should be the first study in the literature to investigate the association of CAR and ISR among these cases.

## Materials and Methods

### Data collection

This was a retrospective, observational research that included 138 consecutive patients who had successful common iliac artery (CIA) or external iliac artery (EIA) stent implantation in a referral tertiary heart center between January 2015 and January 2018. Our institution is a referral cardiovascular center with a high-volume of peripheral interventions (>300 per year), which are mostly performed by interventional cardiologists. In the study, patients with life-limiting claudication despite guideline-mediated medical and exercise therapy due to iliac artery disease undergo iliac artery intervention. Our institution has a detailed electronic recording system of the patients with PAD. Hence, subjects with incomplete data were not included in this analysis. In the present study, subjects who were on chronic renal replacement therapy and who had a diagnosis of acute coronary syndrome in last six months, chronic heart failure, permanent or paroxysmal atrial fibrillation, overt and/or active systemic organ disease, active or chronic infection, malignancy, end-stage diseases were not included. Moreover, patients who had overlapping stents were not enrolled in order to preclude confounding bias. All of the data, including the demographic and clinical characteristics of the subjects as well as interventional procedures were retrieved from the hospital’s electronic medical files. The severity of claudication was assessed according to the Fontaine classification and baseline characteristics of aortoiliac lesions were defined regarding to the Trans-Atlantic InterSociety Consensus II (TASC II) classification before the index procedure.

### Interventional treatment procedure

All procedures were performed in an elective fashion. All of the patients were on a regular dose of daily 100 mg aspirin, 75 mg clopidogrel and statin medication before the procedure. After discharge, all subjects were prescribed at least one month of clopidogrel 75 mg daily, and lifelong 100 mg aspirin daily in addition to having guideline-based medical therapy and supervised exercise. In regards to interventional treatment options, lesions (occlusion or stenosis) shorter than 5 cm are usually treated with percutaneous endovascular interventions (stenting). More complex, particularly TASC II type D lesions, are generally consulted with cardiovascular surgeons for surgical treatment options. However, if the patient refuses to undergo surgery or in cases of high surgical risk, we offered an endovascular therapy as a last resort after carefully discussing the risks and benefits of the procedure. Additionally, given our excellent collaboration with cardiovascular surgeons in our institution, we do have the option of hybrid procedures for the iliac lesions extending to the common femoral artery (CFA). Following the abovementioned approach, only 8.7 % of cases had TASC II type D lesions in our study. Interventional strategies included both ipsilateral and contralateral access to the lesions. The CFA was commonly accepted as a main access location even though the left brachial artery cannulation was performed in case of needed. Primary stenting was the primary choice of therapy, as recommended in the recent North American PAD guidelines.^8^ In the study, the median implemented stent length was 60 mm. Even though balloon-expandable stent platform (Express LD Premounted Stent System, Boston Scientific, USA) was used to treat ostial CIA lesions, we used both self-expandable (Epic Vascular Self-Expanding System, Boston Scientific, USA) and balloon-expandable stent platforms to treat EIA and non-ostial CIA lesions. Whenever a self-expandable stent was preferred, the stent with the size of 1-2 mm larger than the largest diameter of the reference vessel was used. Intravenous unfractionated heparin was administered during all procedures (100 units/kg), and additional doses were applied to achieve activated clotting time between 250 and 350. Our general approach while crossing chronically occluded long segments is to maintain the wires as intraluminal as much as we can. In case of creating a sub-intimal vessel dissection while entering the occlusion, we try to maintain wire loop not bigger than the diameter of the native vessel, keeping in mind that widening of the loop tip means a more extensive dissection, resulting a lower chance of re-entry to the distal vessel true lumen. To prevent this unfavorable situation, we push the support catheter to catch up over the unlopped part of the wire and then pull the wire back to straighten the tip.

### Follow-up and definition of ISR

After a successful revascularization procedure, all patients were regularly evaluated at 1, 6, 12 months, and then every 6-12 months with duplex ultrasonography (DUS) even they were asymptomatic. Patients with recurrent claudication at any time underwent DUS, and further evaluation with peripheral digital subtraction angiography (DSA) was performed. ISR was accepted as >2.4 of the peak systolic velocity index (PSVI) by duplex scan at the target lesion and/or >50% obstruction of the iliac stent by DSA.

### Laboratory analysis

The blood samples, including serum albumin, creatinine, glucose, CRP, and other hematologic parameters, were obtained from all patients in the morning before the procedure. Serum CRP and serum albumin levels were estimated by using the biochemistry auto-analyzer (Roche Cobas 6000, Indianapolis, USA), respectively. In our institution, the normal range for CRP and serum albumin values are 0-5 mg/L and 3.5-5.5 g/dL, respectively. The CAR was determined by dividing serum CRP by serum albumin.

### Statistical analysis

The data were demonstrated as a mean ± SD for parametric or a median [interquartile range (IQ)] for non-parametric variables. The number and percentages were used for categorical variables. The Kolmogorov-Smirnov statistics was applied to assess continuous variables for the normal distribution assumption. The sample t test was used to evaluate the differences between ISR (+) and ISR (-) subjects. Either Pearson’s χ^2^ test or Fisher’s exact test was performed for categorical variables. Receiver operating curve (ROC) was utilized to describe the values of the CAR to predict ISR. Kaplan-Meier curves were performed with using these cut-off values of the CAR, and the Log-rank test was used to compare these groups. The Cox regression analysis was performed to determine the predictors of ISR. The variables with *P* value of <0.10 were included in a Cox regression analysis. In addition, multicollinearity analysis was used for the parameters in the regression model to prevent multicollinearity. *P* values of <0.05 were described as statistically significant. Statistical Package for Social Sciences software (SPSS 22.0 for Windows) was used to carry out all statistical analyses.

## Results

The present study included 138 patients who had successful iliac artery stent implantation due to atherosclerotic involvement of the iliac segment. The clinical and laboratory properties of all cases were displayed Table 1. The mean age of the cases was 61.5 ± 8.8 years, and most cases were male (81.2%). The study population was categorized into two groups; patients with ISR and those without ISR during follow-up. ISR was observed in 40 (28.9%) out of 138 patients in our cohort. We observed that female patients had elevated risk of ISR during long-term period (*P* < 0.05). The frequency of hypertension, diabetes, a history of coronary artery disease, dyslipidemia, and smoking were similar for each group (*P* > 0.05 for all). The patients who had ISR had higher Fontaine stage and TASC II-D lesion class on admission when compared to those who did not suffer from ISR. Not surprisingly, the mean stent length was higher in the ISR (+) group. In terms of laboratory findings, HbA_1c_ levels and the CAR levels on the day of intervention were not indifferent (*P* < 0.01 for both). The other laboratory findings were indifferent for each group (*P* > 0.05 for all). There was a significant similarity between the groups in terms of medications prescribed at discharge. The mean follow-up period after stenting was also similar.

**Table 1:** Clinical and laboratory properties of all patients at baseline

	**Overall (n=138)**	**ISR (-) (n=98)**	**ISR (+) (n=40)**	**P value**
Age, y	61.5 ± 8.8	60.6 ± 8.9	63.9 ± 8.2	0.04
Male, n (%)	112 (81.2)	84 (85.7)	28 (70)	0.03
BMI, kg/m^2^	24.5 ± 2.8	24.2 ± 2.6	24.5 ± 2.7	0.74
Hypertension, n (%)	107 (77.5)	75 (76.5)	32 (80)	0.65
Diabetes mellitus, n (%)	77 (55.8)	51 (52)	26 (65)	0.16
Dyslipidemia, n (%)	101 (73.2)	69 (70.4)	32 (80)	0.24
Smoking, n (%)	99 (71.7)	68 (69.4)	31 (77.5)	0.33
CAD, n (%)	90 (65.2)	62 (63.3)	28 (70)	0.45
**Fontaine classification, n( %)**				
Stage II	85 (61.6)	67 (68.4)	18 (45)	0.02
Stage III	44 (31.9)	28 (28.6)	16 (40)	0.19
Stage IV	9 (6.5)	3 (3.1)	6 (15)	**0.01**
**TASC II lesion classification, n( %)**				
Type A	53 (38.4)	40 (40.8)	13 (32.5)	0.36
Type B	39 (28.3)	33 (33.7)	6 (15)	0.03
Type C	34 (24.6)	23 (23.5)	11 (27.5)	0.61
Type D	12 (8.7)	2 (2)	10 (25)	**< 0.01**
Stent diameter, mm	8.1 ± 1.03	8.2 ± 1.1	8.1 ± 1.1	0.90
Stent length, mm	60 [61]	57 [41.2]	104 [59.7]	**< 0.01**
**Laboratory parameters**				
Hemoglobin, mg/dL	13.1 ± 1.6	13.2 ± 1.7	12.8 ± 1.6	0.29
Leukocyte, x10^9^/L	6.87 ± 1.76	6.84 ± 1.78	7.10 ± 1.74	0.41
Platelet, x10^9^/L	241.6 ± 39.2	236.6 ± 37.3	247.1 ± 41.6	0.45
MPV, fL	8.4 ± 1.5	8.3 ± 1.6	8.7 ± 1.2	0.20
eGFR, mL/min/1.73 m^2^	86.9 ± 16.4	88.4 ± 16.2	83.4 ± 16.5	0.10
HbA_1c_, (%)	7.48 ± 1.95	6.7 ± 1.4	9.2 ± 1.9	**< 0.01**
CRP/Albumin Ratio (CAR)	0.16 [0.57]	0.11 [0.11]	0.90 [0.58]	**< 0.01**
Follow-up time, month	24.7 ± 11	24.1 ± 10.3	26.4 ± 12.5	0.25

BMI, body mass index; CAD, coronary artery disease; CRP, C-reactive protein; eGFR, estimated glomerular filtration rate; ISR, in-stent restenosis; MPV, mean platelet volume; TASC, Trans-Atlantic Inter-Society Consensus.

**Figure 1:**
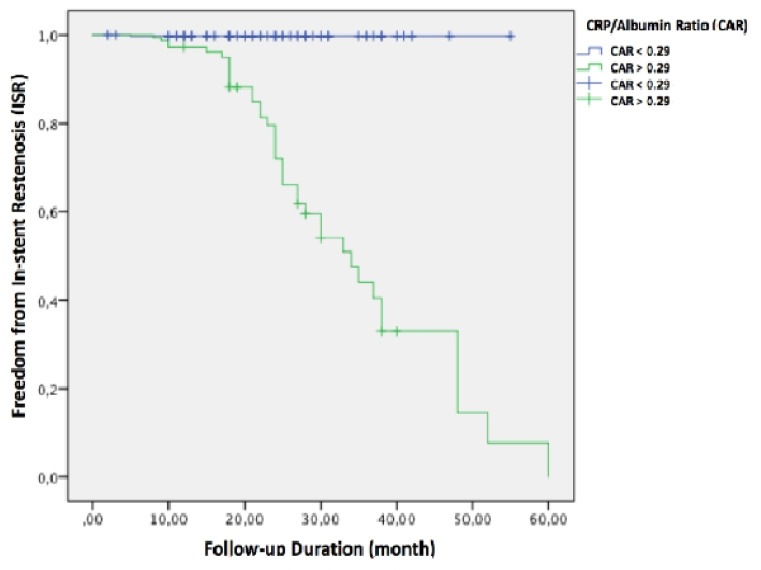
The Kaplan-Meier curve analysis for in-stent restenosis based on the C-reactive protein/albumin ratio values

In univariable Cox regression analysis; Fontaine stage IV symptoms, TASC II-D lesion type, stent length, HbA_1c_ levels, and the CAR were related with ISR. After the inclusion of these variables into the multivariable Cox regression analysis; the CAR (HR: 2.66, 95% CI: 1.66-4.25, *P* < 0.01), stent length (HR: 1.01, 95% CI: 0.99-1.02, *P* = 0.04), and HbA_1c_ levels (HR: 1.22, 95% CI: 0.99-1.51, *P* = 0.04) were found to be independently associated with ISR (Table 2). The variance inflation factor (VIF) as well tolerance factor was performed to access multicollinearity for variables included in the model because all values of tolerance were >0.1 and VIF were <10, respectively. We assumed that there was no multicollinearity between each parameter in the model. In ROC analysis, the CAR value of >0.29 was found to be the optimal value to predict ISR (sensitivity 97.5% and specificity 88.8%, AUC 0.94, *P* < 0.01). In Kaplan-Meier curves, patients with CAR value above the optimal value had a significantly elevated risk for ISR (Log-rank *P* < 0.01) (Figure 1).

**Table 2:** Independentpredictors of in-stent restenosis in multivariable analysis

	**HR (95% CI)**	***P*** **value**
Fontaine stage IV symptoms	0.82 (0.47 – 3.12)	0.68
TASC II type D lesion	1.52 (0.57 – 4.06)	0.40
HbA_1c_	1.22 (0.99 – 1.51)	0.04
Stent length	1.01 (0.99 – 1.02)	0.04
CRP/albumin ratio (CAR)	2.66 (1.66 – 4.25)	< 0.01

CI, confidence interval; CAR, C-reactive protein/Albumin ratio; HR, hazard ratio; ISR, in-stent restenosis; TASC, Trans-Atlantic Inter-Society Consensus.

## Discussion

Our study demonstrated that in PAD patients whose iliac artery involvement was treated with stenting, the stent length, preprocedural HbA_1c_ levels, and the CAR were independently linked with ISR during the long-term period.

Aorto-iliac segment is one of the most common sites of chronic atherosclerotic involvement, and aortoiliac atherosclerosis was founded to be more progressive when compared with infra-inguinal atherosclerosis.^9^ In the current era, open surgical and endovascular treatment approaches constitute the main therapy options for AIOD. Over the last decades, endovascular therapy has continuously developed, and it can, therefore, be applied as an excellent treatment strategy for more types of AIOD. It is associated with not only faster recovery times and shorter hospital stays but also linked with decreased mortality and morbidity as well as improved quality of life when compared with open surgical techniques.^10^

ISR, which can be defined as the gradual re-narrowing of a stented artery due to arterial damage and subsequent neointimal tissue proliferation, is closely associated with systemic inflammation and endothelial dysfunction. Therefore, ISR remains as a significant drawback of percutaneous interventions both in coronary and peripheral vascular beds.^11^ ISR after percutaneous coronary interventions is mostly triggered by endothelial injury during balloon inflation and stent implantation. This vascular damage causes the release of mediators, resulting in the adhesion of monocytes, thrombocytes, and neutrophiles. Mitogenic, thrombogenic, lymphocytic, and vasoactive substances released by these blood cells lead to vasoconstriction, neointimal proliferation, thrombosis, and inflammation that eventually result in ISR.^3,12^ These pathophysiological mechanisms can be assumed for ISR in peripheral vascular beds, as shown by Bleda et al. In this research, the investigators showed that basal levels of inflammatory markers were related with an elevated number of early reintervention following endovascular therapy of PAD.^13^

CRP is the most well-known and investigated inflammatory marker in the current literature. Several clinical studies have shown a strong and independent relationship between higher values of CRP and poor prognosis in cases with cardiovascular disease.^14,15^ In addition to its role in prognosis, Wasser et al found thatsome inflammatory markers, including CRP, play a pivotal role in the occurrence of ISR in subjects who underwent carotid artery stenting.^16^ Moreover, Stone et al showed that preprocedural high sensitivity-CRP values were associated with major adverse limb events in which receiving interventions distal to the iliac arteries.^17^

Hypoalbuminemia usually results from either malnutrition or inflammation or cachexia or all of them. A prior clinical study revealed a significant relationship between lower serum albumin levels and adverse cardiovascular outcomes in subjects with acute myocardial infarction.^18^ Also, it has been demonstrated that lower serum albumin levels are related to coronary ISR.^5^ In terms of the PAD spectrum, Ishii et al found that lower albumin levels could strongly predict major adverse limb events, including target vessel revascularization and amputation.^19^

The CAR, which is a novel inflammation-based prognostic biomarker, can be easily obtained by dividing serum CRP by serum albumin level. The CAR has been extensively investigated in patients with several types of malignancies in previous studies.^20-22^ This new index has also gained attention in cardiovascular disease because studies reported that a combination of these parameters into a single index is a more sensitive parameter in predicting inflammation than CRP or albumin alone due to opposite directions of each marker.^14,23,24^ In addition, it can be hypothesized that as a single inflammatory index, the CAR may provide stability against serum CRP and albumin alone. In accordance with these assumptions, the need for a more reliable indicator than currently used markers for predicting ISR in peripheral artery stenting is obvious. This gap in the literature prompted us to investigate the potential relation of the preprocedural CAR with ISR in the iliac territory. To the best of our knowledge, this should be the first research in this context. Based on our results, we thought that increased preprocedural CAR is closely linked with iliac artery ISR during the long-term follow-up. We considered that this is an important finding because it supports our hypothesis concerning the need for a more sensitive indicator than CRP or albumin alone to predict ISR in the iliac territory. Thus, it may be reasonable to prescribe higher doses of statin and to arrange a more frequent follow-up to patients with a preprocedural CAR > 0.29.

Besides CAR, we noted that the mean stent length and poor glycemic control were independent predictors of ISR. These latter findings of our study were not surprising, which were in accordance with the current literature.

The research had the following limitations. Firstly, it had a retrospective, observational nature; but, all consecutive patients were analyzed in this study. Secondly, our study was conducted in a limited number of PAD cases. Thirdly, despite all subjects were prescribed guideline-based medical therapy and lifestyle modifications after stent implantation, we did not have detailed data with respect to the adherence to these prescriptions and life-style modifications during the long-term follow-up, which could constitute potential confounders for ISR. Fourthly, in our study, we did not collect the data regarding functional outcomes of the patients following an index procedure. Fifth, since the objective of the study was to evaluate the association between ISR and CAR, we did not compare the baseline complexity of the PAD with CAR in our study. Finally, further multi-center, prospective, and larger studies should be performed to illuminate the exact role of the CAR to predict ISR in patients undergoing iliac stenting.

## Conclusion

The present research findings revealed that the CAR as a useful, simple, conventionally available, and inexpensive indicator may be used to estimate the risk of ISR in cases with iliac artery stenting.

## Competing interests

None to declared.

## Ethical approval

The Institutional Review Board approved the design of the current study (28001928-604.01.01). After that, the study was performed according to the principles of the Declaration of Helsinki. Informed consent was not obtained due to the retrospective nature of the study.

## Funding

None.
